# ZnO Nanocomposites Modified by Hydrophobic and Hydrophilic Silanes with Dramatically Enhanced Tunable Fluorescence and Aqueous Ultrastability toward Biological Imaging Applications

**DOI:** 10.1038/srep08475

**Published:** 2015-02-16

**Authors:** Shuying Li, Zongzhao Sun, Rui Li, Minmin Dong, Liyan Zhang, Wei Qi, Xuelin Zhang, Hua Wang

**Affiliations:** 1 Shandong Province Key Laboratory of Life-Organic Analysis, College of Chemistry and Chemical Engineering, Qufu Normal University, Qufu 273165, P. R. China; 2 College of Sport Science, Qufu Normal University, Qufu 273165, P. R. China

## Abstract

Multicolor ZnO quantum dots (QDs) were synthesized and further modified with hydrophobic hexadecyltrimethoxysilane (HDS) and then hydrophilic aminopropyltriethoxysilane (APS) bilayers, resulting in amine-functionalized ZnO@HDS@APS nanocomposites with tunable fluorescence from blue to green yellow. Systematic investigations verify that the resulting ZnO@HDS@APS could display extremely high stability in aqueous media and unexpectedly, dramatically-enhanced fluorescence intensities, which are about 10-fold higher than those of bare ZnO QDs. The feasibility of the as-prepared ZnO nanocomposites for blood, cell, and tissue imaging was preliminarily demonstrated, promising the wide bio-applications for cell or tissue imaging, proteome analysis, drug delivery, and molecular labeling.

Nanostructured ZnO materials have received increasing applications as photocatalysts, transducers, and optical devices^1^. Especially, ZnO nanomaterials with tunable fluorescence are cheap and nontoxic quantum dots (QDs) that possess the potential of replacing the toxic semiconductor QDs (i.e., CdSe and CdTe) to be applied in the optical fields like light-emitting diodes^2^,^3^,^4^ and anti-counterfeit codes^5^, and especially the biological fields such as the biological wastewater treatment^6^, cell imaging or cytotoxicity^7^,^8^, antibacterial^9^,^10^, drug delivery^11^,^12^, and molecular labeling^13^. Nevertheless, colloidal ZnO QDs prepared by the common sol-gel route are unstable and tend to aggregate by undergoing the Ostwald ripening^14^,^15^, showing considerably low quantum yields (QYs). More seriously, the fluorescence of ZnO QDs might be sharply quenched in the aqueous dispersions and the biological media^16^. The resulting growth of ZnO QDs might additionally bring the luminescent shifts, making it a problem in the acquisition of multicolor ZnO QDs^17^,^18^. Therefore, how to enhance the aqueous stability and fluorescence intensity of ZnO QDs is an attractive but very challenging issue to be addressed before they can be used on a large scale in the optical and biological fields.

Recent decades have witnessed the emergence of many surface modification strategies, in which low molecular weight agents, surfactants, polymers, and silanes were employed to obtain stable and intense fluorescence of ZnO QDs^16^,^19^,^20^,^21^,^22^,^23^,^24^,^25^,^26^,^27^,^28^,^29^,^30^,^31^. For example, colloidal ZnO QDs were stabilized in the oleic acid matrix showing the blue mono fluorescence only^19^, which might not be suitably applied in the biological backgrounds with strong protein-sourced blue fluorescence. Various surfactants have also been utilized for the surface modification of ZnO QDs to inhibit their growth in emulsion^20^,^21^,^22^,^23^. Nevertheless, the resulting products might not be stable enough in biological media in addition to the surfactant biotoxicity. A series of polymer-stabilized ZnO QDs have been synthesized for cell imaging^24^, proteome analysis^25^, and drug delivery^26^. These ZnO nanocomposites, however, might suffer from some problems regarding the product purification or re-dispersion in other matrices for further applications^27^,^28^. Particularly, the thick polymer modifiers might induce a great decrease in their fluorescence^29^. Moreover, the silane-based surface modifications of ZnO QDs have been widely proposed^16^,^30^,^31^. Unfortunately, most of the silanized products could exhibit a stable luminescence only in non-aqueous media or organic solution^16^,^30^. Factually, when we utilized aminopropyltriethoxysilane (APS) to coat ZnO QDs, to our surprise, the luminescence of the yielding ZnO@APS could be quenched even faster than that of bare ZnO QDs in water, especially the higher silane dosages were applied.

Inspired by the hydrophobic effects of lotus leaf, in the present work, we seek to first introduce a hydrophobic silane layer of hexadecyltrimethoxysilane (HDS) onto multicolor ZnO QDs, which were initially synthesized by the ultrasonic chemistry with varying [LiOH]/[Zn^2+^] ratios, so as to shield them from the aqueous penetration. Furthermore, a hydrophilic silane layer of APS was modified to improve their aqueous dispersion, resulting in several kinds of functionalized ZnO@HDS@APS with tunable multicolor fluorescence. Importantly, the so prepared ZnO nanocomposites could present the high aqueous stability and unexpectedly, dramatically enhanced fluorescence intensities comparing to bare ZnO QDs. The topological changes and optical performances of the prepared ZnO@HDS@APS in ethanol and water were systematically investigated versus ZnO QDs by using transmission electron microscopy (TEM) imaging, X-ray diffraction (XRD), infra-red (IR) spectra, UV-vis spectra, and fluorescence spectra. Subsequently, the feasibility of the biological applications of ZnO@HDS@APS for the blood, cell, and tissue imaging has been preliminarily demonstrated.

## Results

**Figure 1:**
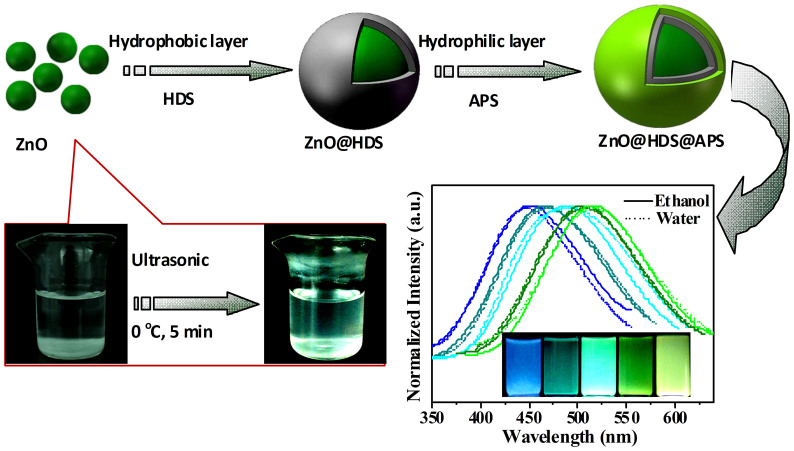
Schematic illustration for the synthesis and bi-silanization modification of multicolor ZnO QDs toward ZnO@HDS@APS nanocomposites with tunable fluorescence using hydrophobic HDS and further hydrophilic APS, where ZnO QDs were prepared by the ultrasonic chemistry at 0°C.

**Figure 2:**
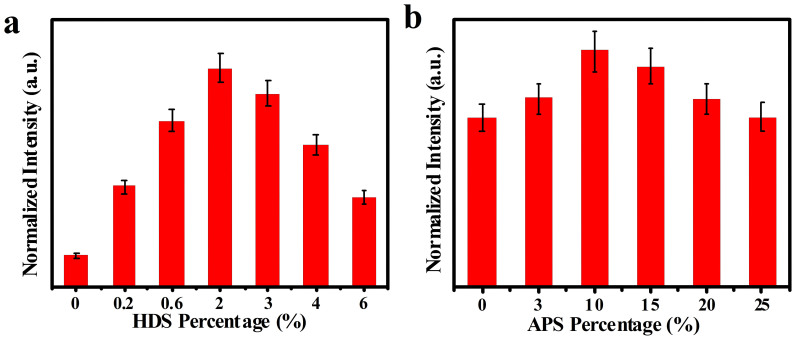
Effects of the bi-silanization dosages of (a) HDS and (b) APS on fluorescence intensities of ZnO@HDS and ZnO@HDS@APS, respectively, which were synthesized in turn in ethanol using different percentages of HDS and APS precursors.

The surface silanization route has been currently employed to modify ZnO QDs, but showing the stable luminescence in non-aqueous media or organic solution only^16^,^30^. Alternatively, here, a bi-silanization modification route was conducted for multicolor ZnO QDs. The typical synthesis and modification procedure was schematically illustrated in Fig. 1. Multicolor ZnO QDs were initially synthesized by the modified sol-gel route of ultrasonic chemistry at 0°C with varying [LiOH]/[Zn^2+^] ratios. Further, they were first modified with hydrophobic hexadecyltrimethoxysilane (HDS) and then hydrophilic APS bilayers, thus yielding functionalized ZnO@HDS@APS nanocomposites with tunable fluorescence from blue to green yellow. Herein, HDS with long alkyl chain could construct the hydrophobic layer on the ZnO QDs to shield them from the water penetration so as to avoid the fluorescence quenching. The enhanced aqueous suspension and bio-modification performances of the resulting ZnO nanocomposites were further obtained by the hydrophilic shell of APS with amine groups. It was found that the florescence intensities of the so prepared ZnO@HDS@APS could depend on the dosages of two silane precursors (see Fig. 2). As can be seen from Fig. 2a, the fluorescence intensities of the ZnO@HDS nanocomposites increase upon forming HDS layer and peak at 2.0% HDS in the mixture, over which the increasing HDS could form thicker HDS layers so as to make the ZnO@HDS more hydrophobic and then precipitate, leading to the quick decrease in the fluorescence intensities measured. Furthermore, the introduction of the hydrophilic APS layer on the ZnO@HDS nanocomposites could significantly improve their aqueous suspension, with the optimal APS dosage being 10% (see Fig. 2b). Notably, the fluorescence intensities of the yielded ZnO@HDS@APS could thus be dramatically enhanced by comparing to bare ZnO QDs in both ethanol and aqueous solution as demonstrated afterwards.

**Figure 3:**
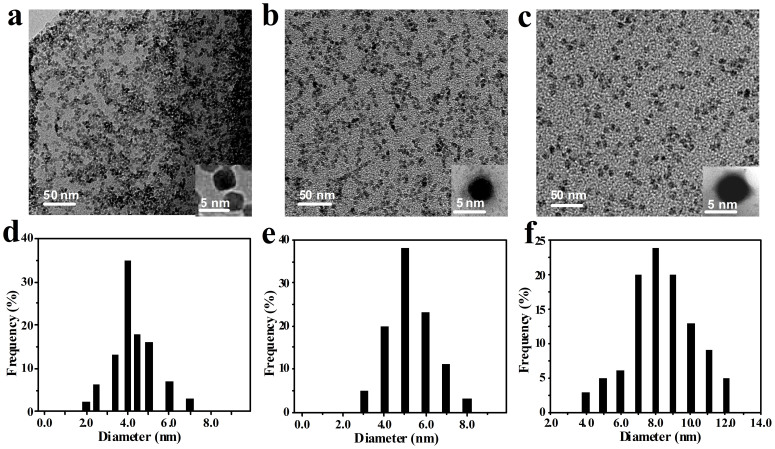
TEM images and hydrodynamic diameter distributions of (a and d) ZnO QDs, ZnO@HDS (b and e), and (c and f) ZnO@HDS@APS nanocomposites, where ZnO cores were prepared at the [LiOH]/[Zn^2+^] ratio of 1.3.

Figure 3 shows the topological characterization of ZnO@HDS@APS nanocomposites by TEM imaging, taking ZnO@HDS and bare ZnO QDs as the controls. One can note that the ZnO QDs (see Fig. 3a), ZnO@HDS (see Fig. 3b), and ZnO@HDS@APS nanocomposites (see Fig. 3c) are uniform and mono-dispersed, showing an average hydrodynamic diameter of about 4.0 nm (see Fig. 3d), 5.0 nm (see Fig. 3e), and about 8.0 nm (see Fig. 3f), respectively, where the increasing sizes could confirm separately the modification of HDS layer and HDS@APS bilayer on the ZnO QDs. The core-shell shape and structure could also be evidenced more clearly from the corresponding amplified particles of ZnO@HDS (Figure 3b, Insert) and ZnO@HDS@APS (see Fig. 3c, Insert). Compared to bare ZnO QDs, the ZnO cores of ZnO@HDS and ZnO@HDS@APS nanocomposites might show little change in size, indicating the well depressed growth of ZnO QDs in the silane matrices.

**Figure 4:**
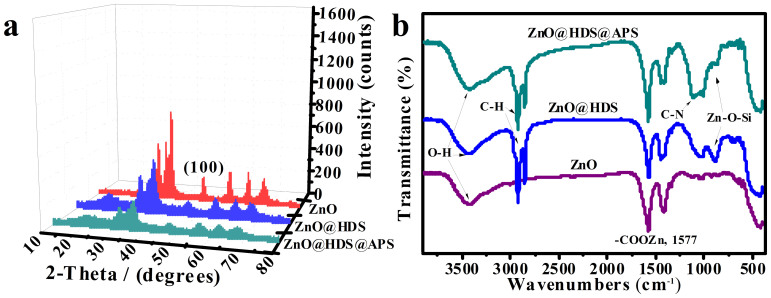
(a) XRD data and (b) IR spectra of ZnO, ZnO@HDS, and ZnO@HDS@APS, of which ZnO QDs were synthesized at the [LiOH]/[Zn^2+^] ratio of 1.3.

Figure 4a depicts the comparison of XRD patterns among ZnO QDs, ZnO@HDS, and ZnO@HDS@APS powders so yielded. Apparently, ZnO in these products can display the typical wurtzite nanostructures as observed elsewhere^32^. The peaks at 22 degrees of ZnO@HDS and ZnO@HDS@APS nanocomposites are assigned to the formation of HDS layer and HDS@APS bilayer on the ZnO QDs. Moreover, little broadened ZnO peak at (110) was found for ZnO@HDS@APS versus ZnO QDs, implying they might share the approximate ZnO crystallite sizes^31^. Accordingly, the bi-silanization could desirably restrict the growth of trapped ZnO QDs to ensure their luminescence stability. Furthermore, IR spectra were recorded to explore the composition of ZnO@HDS@APS (see Fig. 4b). As expected, the peaks of C-H vibration (2850–3000 cm^−1^) and Zn–O–Si symmetrical stretching vibration (870 cm^−1^) indicate the polysiloxane shelled on ZnO QDs^33^. Meanwhile, the peaks at 1000–1100 cm^−1^ and 3300–3600 cm^−1^ are assigned to the C-N vibration of amine groups of APS and the O-H vibration of hydroxyl group on ZnO QDs, respectively. The above results confirm the successful formation of ZnO@HDS and ZnO@HDS@APS nanocomposites.

**Figure 5:**
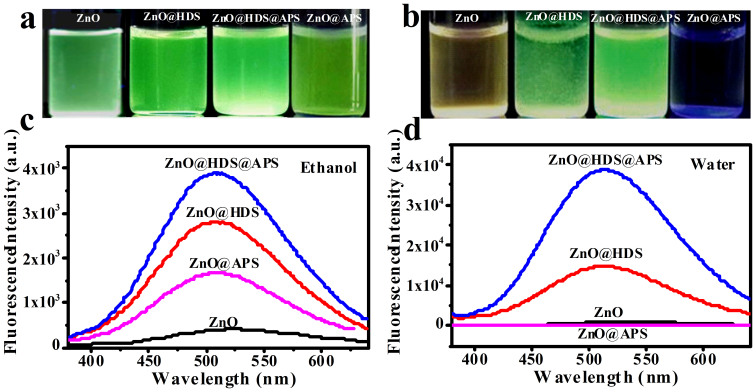
Silanization effects on ZnO luminescence by digital photographs (UV light at 302 nm) and the fluorescence spectra of ZnO, ZnO@HDS, ZnO@HDS@APS, and ZnO@APS in (a and c) ethanol and (b and d) water. ZnO QDs were synthesized at the [LiOH]/[Zn^2+^] ratio of 1.3.

Moreover, the bi-silanization-induced enhancement in the suspension stability and luminescence performances of ZnO@HDS@APS was comparably investigated, where ZnO QDs synthesized at [LiOH]/[Zn^2+^] ratio of 1.3 were employed as an example of multicolor ZnO QDs to be shelled separately with the hydrophobic HDS layer and/or hydrophilic APS layer. Figure 5 describes the comparison of fluorescence emissions among the resulting ZnO QDs, ZnO@HDS, ZnO@HDS@APS, and ZnO@APS suspended separately in ethanol and water. As can be seen from Fig. 5a, all the silanized products in ethanol could display the luminescent emissions brighter than that of ZnO QDs. However, when these ZnO nanomaterials were dispersed into water, the fluorescence quenching could occur in distinctly different degrees (see Fig. 5b). Herein, the fluorescent emissions of ZnO@HDS@APS and ZnO@HDS in water were highly stable with no significant emission change, whereas those of ZnO QDs and ZnO@APS could sharply decay till being completely quenched. Interestingly, the fluorescence ZnO@APS in water was quenched even faster than that of bare ZnO QDs, presumably due to that the aqueous alkalinity of amine-derivatized hydrophilic APS layers might gradually etch ZnO cores and/or booster the penetration of the fluorescence-quenchable water into the ZnO cores. In contrast, the hydrophobic HDS layers on the ZnO@HDS@APS and ZnO@HDS might curb the water penetration from quenching the ZnO fluorescence. Notably, the formation of HDS layers on the ZnO QDs could make the resulting ZnO@HDS hydrophobic so as to exhibit a poor dispersion in water due to the presence of the long alkyl chain of HDS (see Fig. 5b). Therefore, the introduction of hydrophilic APS layer onto ZnO@HDS to yield ZnO@HDS@APS could greatly improve their aqueous suspension. To demonstrate that, a quantitative comparison of fluorescence spectra was further performed among these nanomaterials dispersed separately in ethanol (see Fig. 5c) and water (see Fig. 5d). As manifested in Fig. 5c, the fluorescence intensities of ZnO@HDS, ZnO@HDS@APS, and ZnO@APS in ethanol are about 7.1, 10.0, and 4.2 times higher than that of bare ZnO QDs, respectively. Obviously, ZnO@HDS@APS could achieve the most enhanced fluorescence intensity. Importantly, Figs. 5b and 5d reveal that the modification of HDS layer on ZnO QDs could help to attain the ZnO fluorescence well stabilized in water. Additionally, the amine-derivatized APS shells of ZnO@HDS@APS might further enhance the ZnO fluorescence by the well known electron-donor effects of amine groups, in addition to the surface functionalization of reactive amine for the biological modifications. Furthermore, the UV-vis spectra of these ZnO products in ethanol and water were comparably studied (see Supplementary Fig. S1 online). One can find that ZnO@HDS@APS in ethanol (see Supplementary Fig. S1a online) and water (see Supplementary Fig. S1b online) showed no obvious shift of absorption peaks, whereas ZnO QDs displayed a red-shift reflecting the increase in particle size. Accordingly, bare ZnO QDs might grow in water, which could be significantly depressed by the bi-silanization once forming the ZnO@HDS@APS. As for ZnO@APS, the ZnO absorption peak could gradually disappear in water, presumably due to the particle etching of ZnO QDs triggered by the alkalinity of the amine-derivatized APS shells in water as aforementioned.

**Figure 6:**
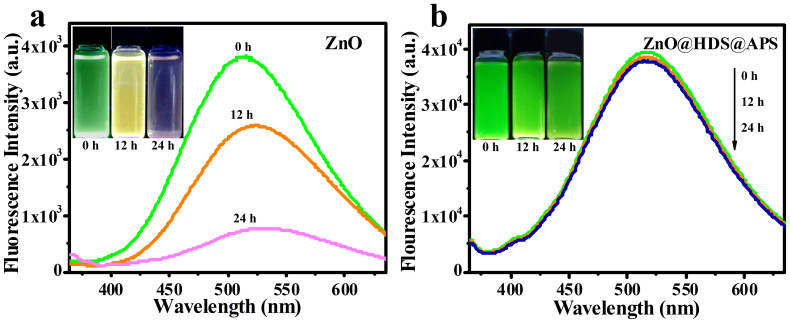
Comparison of suspension stability and anti-photobleaching ability by the digital photographs and fluorescent emission spectra between (a) ZnO and (b) ZnO@HDS@APS in ethanol, separately exposed under UV xenon lamp for different time intervals of 0, 12, and 24 h.

The anti-photobleaching stability of ZnO@HDS@APS in ethanol were investigated in comparison to bare ZnO QDs (see Fig. 6). It is observed that fresh ZnO QDs could exhibit the green emission (see Fig. 6a). With continuous irradiation under the strong UV xenon lamp, however, they could dramatically decay companying with the aggregation, with the luminescent colors changing from green to light pink within 24 h. Meantime, the fluorescence emission of bare ZnO QDs could largely decrease with a red-shift in the emission peaks, suggesting that the size of ZnO QDs might increase as the time underwent, companied by the decrease in their fluorescence intensities. By comparison, ZnO@HDS@APS could well sustain their bright emission (see Fig. 6b). Also, their corresponding emission spectra could keep the extremely identical profiles over the aging period, with no obvious change in the fluorescence intensities. The above data are in well consistent with their fluorescence excitation spectra in ethanol (see Supplementary Fig. S2 online). Accordingly, the so prepared ZnO@HDS@APS nanocomposites could attain suspension ultrastability and high anti-photobleaching ability in ethanol versus ZnO QDs. Moreover, the condition-against performances of ZnO@HDS@APS in water were systematically examined, comparing to bare ZnO QDs (see Supplementary Fig. S3 online). It is witnessed that the fluorescence intensities of ZnO@HDS@APS and ZnO QDs in water could depend on the testing conditions including temperatures, pH values, ionic strengths, and storage time. For example, ZnO@HDS@APS could be pH-sensitive and would be more stable in the neutral range (see Supplementary Fig. S3b online). This interesting phenomenon indicates that ZnO@HDS@APS might be employed to carry or encapsulate the meaningful drugs for fluorescence-trackable and pH-sensitive drug delivery in the cell biology. Moreover, the fluorescence of ZnO@HDS@APS could mostly survive in the aqueous conditions up to 340 K (see Supplementary Fig. S3a online), 0.20 mol L^−1^ NaCl (see Supplementary Fig. S3c online), and five-month storage (see Supplementary Fig. S3d online). With respect to bare ZnO QDs, their fluorescence could be basically quenched under the harsh conditions.

**Figure 7:**
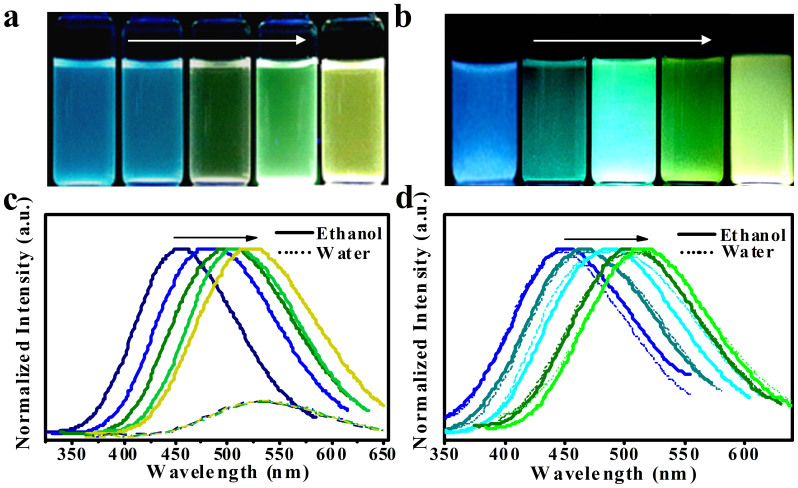
Comparison of luminescence properties among (a) ZnO and (b) ZnO@HDS@APS with five tunable luminescent colors in ethanol, and the fluorescence emission spectra of (c) ZnO and (d) ZnO@HDS@APS separately in ethanol (solid lines) and water (dotted lines). Multicolor ZnO QDs were separately synthesized at different [LiOH]/[Zn^2+^] ratios of 2.5, 2.1, 1.7, 1.3, and 1.0 (from left to right).

Subsequently, the developed bi-silanization strategy was employed for the modification of several kinds of multicolor ZnO QDs, which were synthesized at varying [LiOH]/[Zn^2+^] ratios of 2.5, 2.1, 1.7, 1.3, and 1.0 in ethanol, resulting in functionalized ZnO@HDS@APS nanocomposites with tunable fluorescence from blue to green yellow (see Fig. 7). Here, the profiles of UV-vis spectra (colors) of the yielded ZnO QDs and ZnO@HDS@APS could depend on the [LiOH]/[Zn^2+^] ratios used (see Supplementary Fig. S4 online), which absorption peaks reflected the difference in particle sizes of ZnO QDs^31^. Figure 7 displays the comparison of luminescence properties among the multicolor ZnO@HDS@APS and corresponding ZnO QDs separately in ethanol and water. As shown in the photographs in Fig. 7a, bare ZnO QDs exhibited five luminescent colors in ethanol, so did the ZnO@HDS@APS (see Fig. 7b) but with much brighter luminescence. Figure 7c shows the fluorescence emission spectra of ZnO QDs, of which the luminescent emissions may range from ca. 461 nm (blue) to ca. 521 nm (yellow) in ethanol (solid lines). However, after exposure in water, their fluorescence could be dramatically quenched with the fluorescence intensities decreased by about 5.0 times (dotted lines). Remarkably, the emissions of ZnO QDs could finally shift to one fluorescent emission of ca. 535 nm, showing the untunable fluorescence properties in water. In contrast, ZnO@HDS@APS could exhibit the tunable luminescence emissions ranging from ca. 446 nm (blue) to ca. 517 nm (green yellow) in ethanol (see Fig. 7d, solid lines). Importantly, their emissions could be highly stable in water with no obvious shift (see Fig. 7d, dotted lines). This evidence was further verified by the corresponding fluorescent excitation spectra (see Supplementary Fig. S5 online). Therefore, the as-prepared multicolor ZnO@HDS@APS nanocomposites could achieve high aqueous stability and especially greatly enhanced tunable fluorescence, thus promising for the extensive applications in the optical and biological fields.

**Figure 8:**
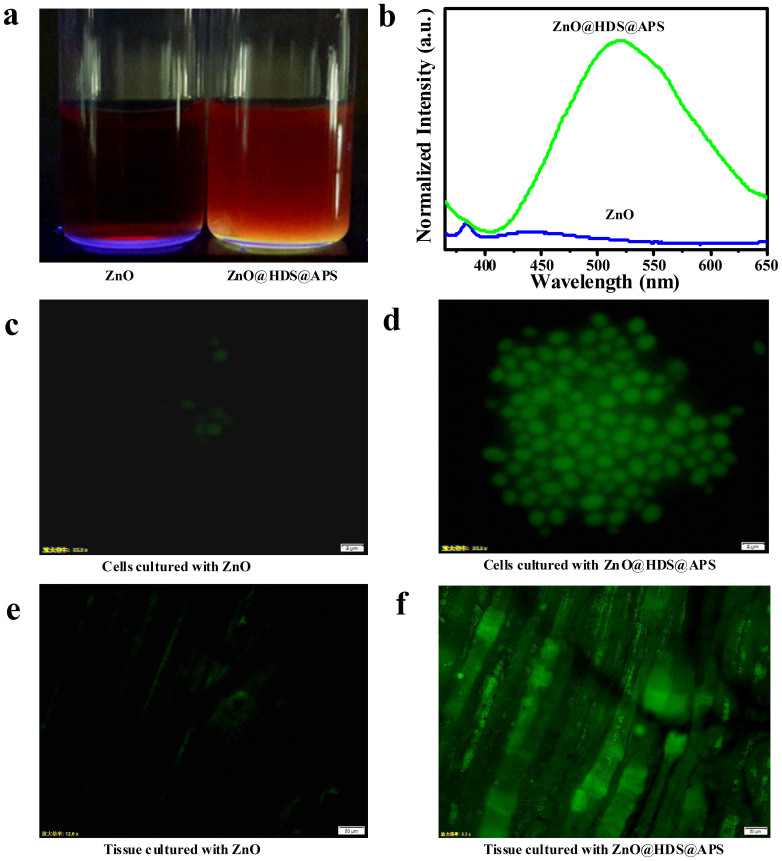
(a) Digital photographs under UV light and (b) the fluorescence intensities of ZnO and ZnO@HDS@APS spiked in blood; the images of fluorescent microscopy for yeast cells cultured separately with the YPD substrate containing (c) ZnO and (d) ZnO@HDS@APS, and for the mouse muscle tissue cultured separately with (e) ZnO and (f) ZnO@HDS@APS.

To access the feasibility of practical applications of these ZnO nanocomposites in the biological fields, ZnO@HDS@APS were utilized separately for the blood, cell, and tissue imaging, taking bare ZnO QDs for comparison (see Fig. 8). The comparison of imaging photographs (see Fig. 8a) and the quantitative fluorescence intensities (see Fig. 8b) were separately conducted between ZnO@HDS@APS and ZnO QDs spiked in blood. Obviously, the amine-functionalized ZnO@HDS@APS probes could enjoy the strong and highly stable fluorescence emission in blood, thus confirming the feasibility for the biological applications like proteome analysis and molecular labeling. Furthermore, ZnO@HDS@APS nanocomposites were introduced for cell and tissue culture by separately using yeast cells and mouse muscle tissues as the testing models. As shown in Fig. 8d, yeast cells so incubated were visible with bright green fluorescence under UV excitation, indicating the effective assimilation of ZnO@HDS@APS, whereas the ones with bare ZnO QDs were almost invisible because their fluorescence might be rapidly quenched in the incubation media (see Fig. 8c). Also, yeast cells could grow into the colonies in the substrate containing high-level ZnO@HDS@APS (data not shown), demonstrating the additional biocompatibility of the amine-derivatized ZnO nanocomposites. Figure 8f illustrates the muscle tissue permeated with strongly fluorescent ZnO@HDS@APS, in contrast to the one with the fluorescence-dimmed ZnO QDs (see Fig. 8e). The above results indicate the amine-functionalized ZnO@HDS@APS with tunable strong fluorescence may meet with the various needs of the biological studies such as blood analysis, cell or tissue imaging, and drug delivery.

In summary, the large-scale optical and biological applications of ZnO QDs can be limited due to they are unstable and tend to aggregate with sharply-quenched luminescence upon aqueous exposure. Inspired by the hydrophobic effect of lotus leaf, herein, multicolor ZnO QDs were initially synthesized and further coated with the hydrophobic HDS and then hydrophilic APS bilayers by a novel bi-silanization modification route. The resulting functionalized ZnO@HDS@APS nanocomposites could present tunable fluorescence from blue to green yellow. Importantly, they could enjoy aqueous ultrastability and surface functionalization and unexpectedly, the dramatically-enhanced fluorescence intensities, which are about 10 times higher than those of bare ZnO QDs. The topological structure and composition changes and optical performances of the as-prepared ZnO@HDS@APS in ethanol and water were systematically demonstrated versus ZnO QDs by using TEM imaging, XRD, IR spectra, UV-vis spectra, and fluorescence spectra. Subsequently, the feasibility of the biological applications of ZnO@HDS@APS for the blood, cell, and tissue imaging has been preliminarily demonstrated. Results indicate that the amine-functionalized ZnO@HDS@APS can promise the potential applications in the biological fields such as molecular labeling, cell or tissue imaging, cytotoxicity, proteome analysis, and drug delivery. Future applications of the fluorescence-tunable ZnO nanocoposites for designing the useful optical devices such as light-emitting diodes and anti-counterfeit codes are undergoing. Such a facile bi-silanization route should also be extended for the modification of varying nanomaterials like a variety of QDs to enhance their fluorescence, surface functionalization, and aqueous stability for the optical and biological applications on a large scale.

## Methods

### Materials and Instruments

Zinc acetate dehydrate (99%), LiOH·H_2_O (99%), acetone (99%), absolute ethanol, and yeast cells were purchased from Sigma-Aldrich (Beijing, China). Yeast peptone dextrose agar (YPD) was obtained from Aladdin. Aminopropyltriethoxysilane (APS, 98%) and hexadecyltrimethoxysilane (HDS, 97%) were bought from Sinopharm Chemical Reagent Co., Ltd (Shanghai, China). Human blood was supplied by the University Hospital (Qufu, China). Deionized water (>18 Mohm) was obtained from an Ultra-pure water system (Pall, USA).

Transmission electron microscopy (TEM, Tecnai G20, FEI, USA) imaging operated at 100 kv were employed to characterize the prepared ZnO QDs and nanocomposites. Fourier transform infrared (Thermo Nicolet Nexus 470FT, USA) spectra and X-ray diffraction (XRD, Mini Flex 600, Japan) patterns of ZnO QDs, ZnO@HDS and ZnO@HDS@APS nanocomposites were recorded. UV-3600 spectrophotometer (Shimadzu, Japan) and Fluorescence spectrometer (Horiba, Fluoro Max-4, Japan) on a slit of 5.0 nm were used to measure the UV-vis spectra and fluorescent spectra of ZnO QDs and nanocomposites, respectively. Research fluorescent inverted microscope (Olympus, IX73-DP80, Japan) was employed to image yeast cells and muscle tissue which were separately cultured overnight with ZnO@HDS@APS or ZnO QDs in the light and dark fields. Anti-photo bleaching investigation of ZnO and ZnO@HDS@APS were observed using a Xenon lamp dives (XL-600, China). The photographs of different reaction products were recorded under UV light at 302 nm. Moreover, Table centrifuge (Thermo Scientific, Deutschland) and Low-temperature (constant-temperature) stirring bath (DHJF-4005, China) were used in the prepared procedures.

### Preparation of ZnO Quantum Dots

ZnO QDs were prepared using the modified by the modified sol-gel route reported by Spanhel and Anderson^32^. In briefly, an amount of zinc precursor was dissolved in 45 mL hot ethanol and then the solution was cooled down to 0°C. An aliquot of LiOH·H_2_O was then added into the mixture at 0°C under ultrasound conditions. Accordingly, multicolor ZnO QDs were separately synthesized at 0°C for 5 min by using different [LiOH]/[Zn^2+^] ratios of 2.5, 2.1, 1.7, 1.3, and 1.0. Furthermore, the resulting ZnO QDs were precipitated by an aliquot of acetone. After that, the products were centrifuged and re-dispersed in ethanol to be washed for several times to remove any unreacted molecules. Subsequently, multicolor ZnO QDs were stored in dark or further dried for the characterization.

### Preparation of ZnO@HDS@APS Nanocomposites

The ZnO@HDS@APS nanocomposites with tunable fluorescence were synthesized separately by coating the fresh multicolor ZnO QDs above first with the hydrophobic HDS layer and then the hydrophilic APS layer by the silane hydrolysis procedure in ethanol. Typically, an aliquot of HDS precursor, with different percentages of 0%, 0.20%, 0.60%, 2.0%, 3.0%, 4.0%, 6.0% in ethanol, were separately introduced into the multicolor ZnO QDs to be stirred 1 h. The as-prepared ZnO@HDS nanocomposites were harvested by centrifugation and then washed by ethanol for several times to remove any redundant reagent, followed by the dispersion in ethanol. Moreover, APS precursor, with different percentages of 3.0%, 10%, 15%, 20% and 25% in the mixtures, was separately added into the ZnO@HDS suspensions to be stirred at the low temperature. Multicolor ZnO@HDS@APS nanocomposites were finally obtained after the centrifugation and washing procedures mentioned above. In addition, ZnO@APS nanocomposites were synthesized accordingly by coating bare ZnO QDs with 10% APS. All of the ZnO nanocomposites were stored in dark or further dried for the characterization.

### Preliminary Biological Applications

An aliquot of ZnO QDs or ZnO@HDS@APS nanocomposites (0.010 mmol L^−1^ Zn^2+^) was separately spiked into fresh whole blood by 20-fold dilution to be incubated for 1 h at 37°C. Their photographs under UV light at 302 nm were taken comparably. Furthermore, the fluorescence spectra of these diluted samples in blood were recorded.

Yeast cells were centrifugated at 8000 rpm for 5.0 min, and then washed with 0.85% NaCl solution. Further, they were diluted and spread on the YPD agar plates to be incubated overnight at 30°C. After the centrifugation and washing for several times, an aliquot of ZnO or ZnO@HDS@APS nanocomposites (0.020 mmol L^−1^ Zn^2+^) was injected, followed by being incubated for 1 h at 30°C. After washing twice, the resulting cell mixtures were separately dropped onto the slides to be imaged separately in the light and dark fields using the fluorescent inverted microscope. Moreover, an aliquot of ZnO or ZnO@HDS@APS (0.020 mmol L^−1^ Zn^2+^) was dissolved in the cell culture reagent with dimethyl sulfoxide (Sigma). Then, the alive muscle tissue from a mouse was cultured in the cell culture reagent to be incubated overnight at 37°C. After washed twice with KHB buffer, the resulting tissue was spread on the slide to be observed using the fluorescent inverted microscope.

## Author Contributions

H.W. conceived the project and designed the experiments. S.L. conducted the main experiments, data analysis, and wrote the paper. Z.S. performed with the synthesis and fluorescence tests. R.L. and D.M. did UV-vis characterization and the anti-photobleaching tests. L.Z. assisted with the TEM and IR characterization. W.Q. and X.Z. contributed to the cell and tissue imaging.

## Supplementary Material

Supplementary Information Revised Supplementary Information
