# Supplementary material for: ZnO Nanocomposites Modified by Hydrophobic and Hydrophilic Silanes with Dramatically Enhanced Tunable Fluorescence and Aqueous Ultrastability toward Biological Imaging Applications
Source: Sci Rep. 2015 Feb 16;5:8475. doi: 10.1038/srep08475 (PMC4329562; doi:10.1038/srep08475)
Supplement: Supplementary Information — Revised Supplementary Information [file srep08475-s1.pdf]

Supplementary Information for

ZnO Nanocomposites Modified by Hydrophobic and Hydrophilic Silanes with Dramatically Enhanced Tunable Fluorescence and Aqueous Ultrastability toward Biological Imaging Applications

Shuying Li,[1] Zongzhao Sun,[1] Rui Li,[1] Minmin Dong,[1] Liyan Zhang,[1] Wei Qi,[1] Xuelin Zhang[2] & Hua Wang[1]*

[1] Shandong Province Key Laboratory of Life-Organic Analysis, College of Chemistry and Chemical Engineering, Qufu Normal University, Qufu, 273165, P. R. China.

[2] College of Sport Science, Qufu Normal University, Qufu, 273165, P. R. China.

* E-mail: huawangqfnu@126.com; Tel: (+86) 5374456306.

List of Contents

Figure S1 UV-Vis absorption profile of ZnO, ZnO@HDS@APS, and ZnO@APS.

Figure S2 Fluorescence excitation profile of ZnO, ZnO@HDS@APS, and ZnO@APS.

Figure S3 Effects of aqueous environmental conditions profile of ZnO and ZnO@HDS@APS.

Figure S4 UV-vis absorption spectra profile of ZnO and ZnO@HDS@APS depends on the [LiOH]/[Zn^{2+}] ratios.

Figure S5 The fluorescence excitation spectra profile of ZnO and ZnO@HDS@APS depends on [LiOH]/[Zn^{2+}] ratios and solvents.

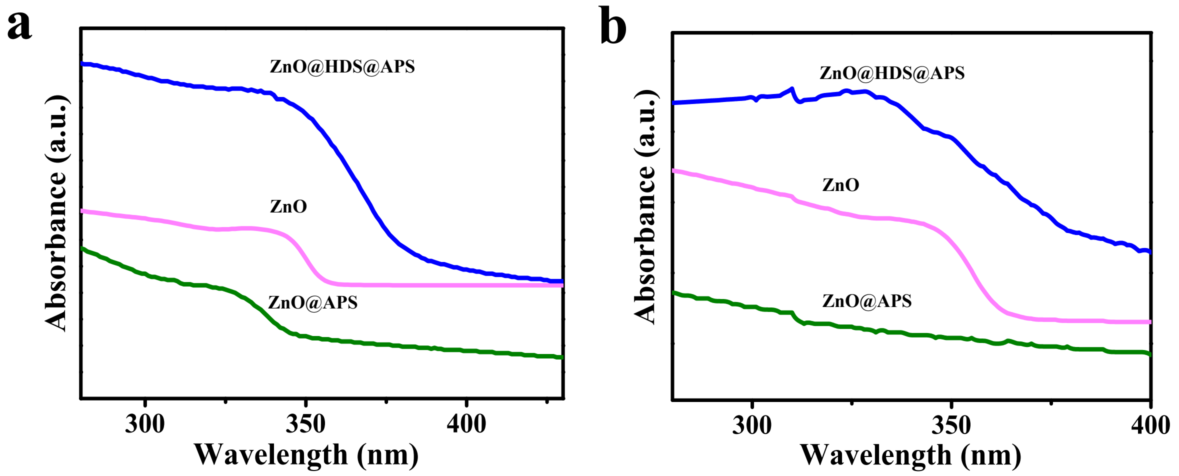

Figure S1. UV-Vis absorption spectra in (**a**) ethanol and (**b**) water for ZnO, ZnO@HDS@APS, and ZnO@APS.

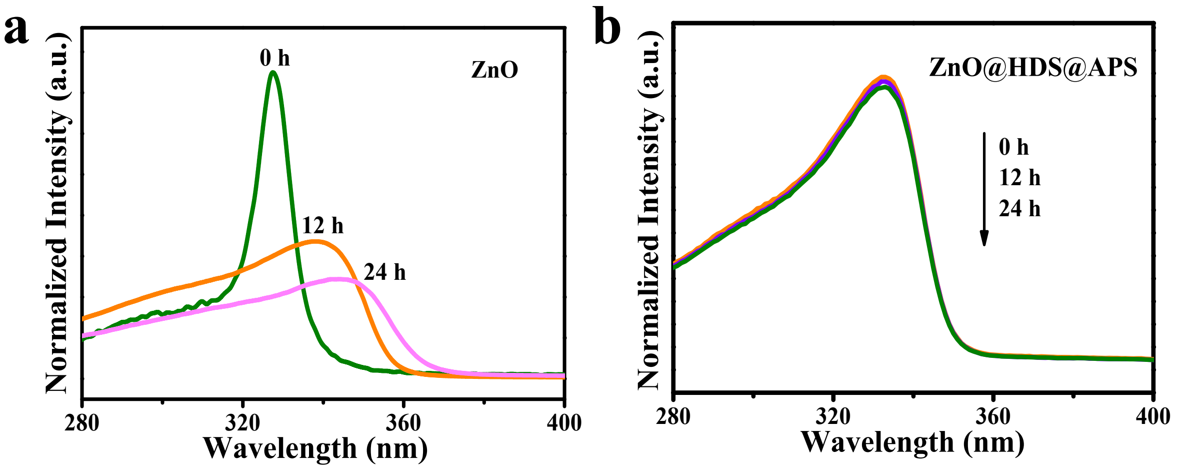

Figure S2. Fluorescence excitation spectra of time-dependent photobleachings for (**a**) ZnO and (**b**) ZnO@HDS@APS, which were exposed under UV xenon lamp for different time intervals of 0, 12, and 24 h.

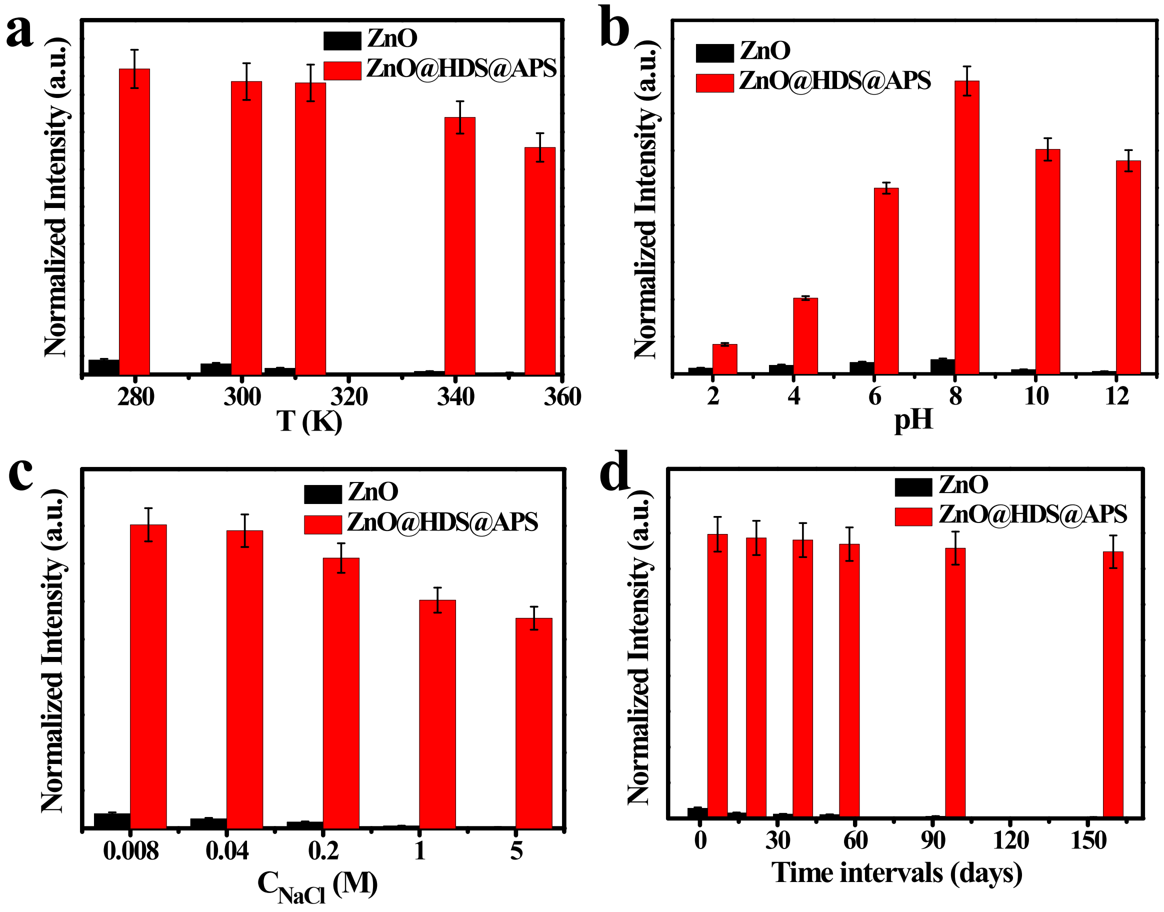

Figure S3. Effects of aqueous environmental conditions of (**a**) temperature, (**b**) pH values, (**c**) ionic strength, and (**d**) storage time on the fluorescence intensities of ZnO QDs and ZnO@HDS@APS, each of which was exposed in the corresponding conditions for 3 h except for (**d**) that was performed at different time intervals indicated.

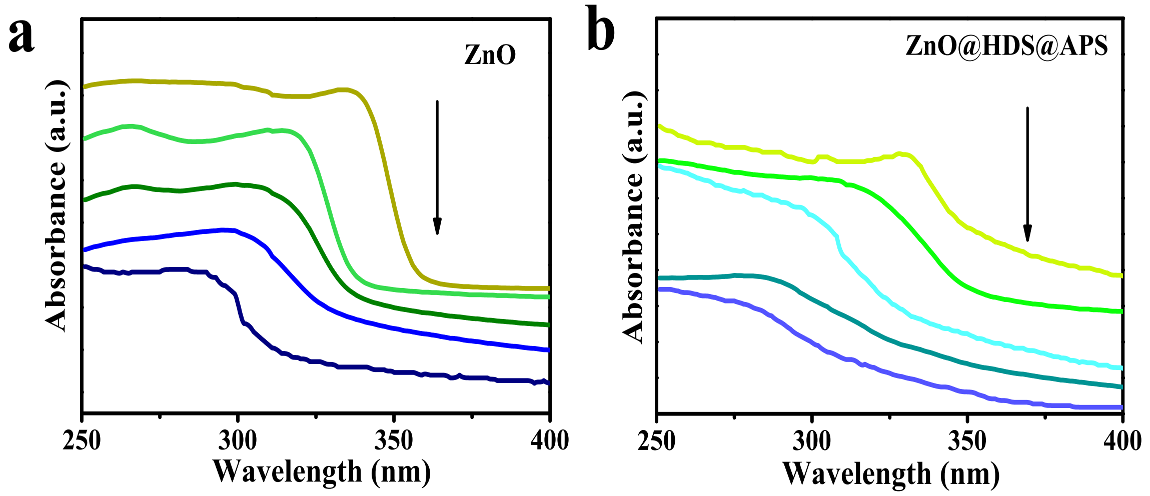

Figure S4. The [LiOH]/[Zn^{2+}] ratios-dependent UV-vis absorption spectra of (**a**) ZnO QDs and (**b**) ZnO@HDS@APS, where ZnO QDs were synthesized in ethanol at varying [LiOH]/[Zn^{2+}] ratios of 2.5, 2.1, 1.7, 1.3, and 1.0 (from up to down).

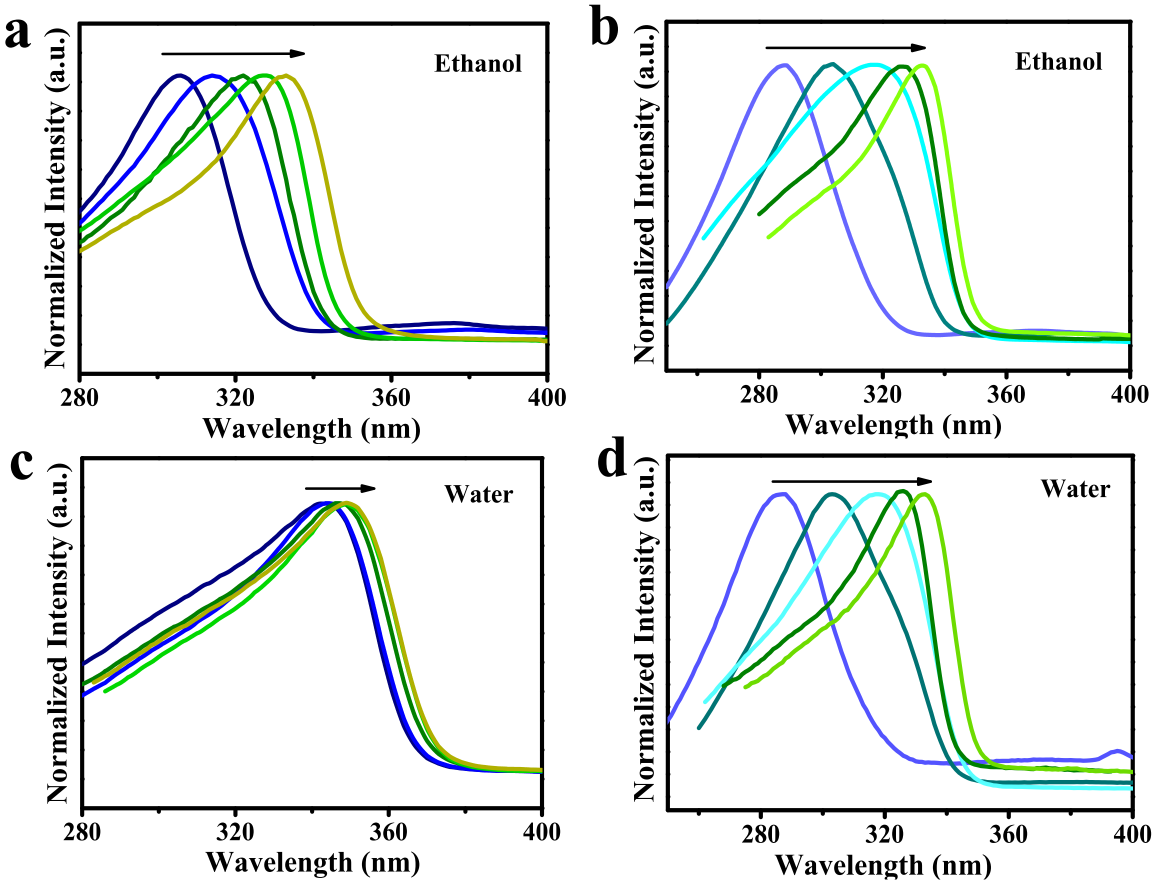

Figure S5. The fluorescence excitation spectra of (**a** and **c**) ZnO and (**b** and **d**) ZnO@HDS@APS with five tunable colors in ethanol and water, of which multicolor ZnO QDs were separately synthesized at different [LiOH]/[Zn^{2+}] ratios of 2.5, 2.1, 1.7, 1.3, and 1.0 (from left to right).
